# Successful thoracic endovascular aortic repair for complicated Stanford type B acute aortic dissection with acute renal failure and vascular remodelling after intervention: case report and 5-year follow-up

**DOI:** 10.1093/ehjcr/ytag336

**Published:** 2026-05-08

**Authors:** Hirotsugu Kurobe, Tomohide Higaki, Takuma Fukunishi, Takashi Nishimura, Hironori Izutani

**Affiliations:** Department of Cardiovascular and Thoracic Surgery, Ehime University Graduate School of Medicine, Shizukawa 454, Toon, Ehime, Japan; Department of Cardiovascular and Thoracic Surgery, Ehime University Graduate School of Medicine, Shizukawa 454, Toon, Ehime, Japan; Department of Cardiovascular and Thoracic Surgery, Ehime University Graduate School of Medicine, Shizukawa 454, Toon, Ehime, Japan; Department of Cardiovascular and Thoracic Surgery, Ehime University Graduate School of Medicine, Shizukawa 454, Toon, Ehime, Japan; Department of Cardiovascular and Thoracic Surgery, Ehime University Graduate School of Medicine, Shizukawa 454, Toon, Ehime, Japan

**Keywords:** Case report, Stanford type B aortic dissection, Thoracic endovascular aortic repair, Vascular remodelling

## Abstract

**Background:**

Surgical treatment such as grafting or thoracic endovascular aortic repair (TEVAR) is recommended for Stanford type B acute aortic dissection (SB-AAD) with complications. We report a case of SB-AAD with acute renal failure caused by malperfusion that was successfully treated with TEVAR.

**Case summary:**

A 69-year-old man was diagnosed with SB-AAD at another hospital. Because he experienced worsening renal function, the patient was referred to our hospital. Enhanced computed tomographic angiography (CTA) revealed an entry point at the distal aortic arch and re-dissection that extended to the level of the bilateral iliac arteries. The entry site was approximately 20 mm distal to the origin of the subclavian artery on the greater curvature side. Because this false lumen compressed both renal arteries, we suspected renal ischaemia as the cause of acute renal failure and oliguria. We performed urgent TEVAR with left subclavian artery occlusion to improve renal ischaemia and function. Thereafter, urine output increased and renal function improved. Follow-up CTA performed 1 week postoperatively showed no enlargement of the dissection cavity or leakage. Favourable vascular remodelling was observed during 5-year follow-up.

**Discussion:**

Thoracic endovascular aortic repair performed in the early phase of SB-AAD may result in a satisfactory clinical course and vascular remodelling. However, careful consideration of its timing is required.

Learning points

- Dynamic true lumen compromise may underlie renal impairment in type B aortic dissection, even without obvious clinical signs of malperfusion signs.
- Restoring true lumen flow improves secondary organ perfusion in type B aortic dissection with malperfusion.
- Early intervention for dynamic compromise can restore adequate organ perfusion and prevent irreversible injury.

## Introduction

Recently, the use of pre-emptive thoracic endovascular aortic repair (TEVAR) for uncomplicated Stanford type B acute aortic dissection (SB-AAD) to prevent late aortic re-dissection or enlargement and improve long-term morbidity and mortality has increased.^1^ However, performing TEVAR for aortic dissection is associated with the risk of complications such as stent-induced re-dissection, which may be more probable depending on the timing of the procedure.^2^ Stanford type B acute aortic dissection is generally managed with strict blood pressure control; however, surgical intervention is indicated for SB-AAD with complications such as rupture or organ malperfusion.^3^ Thoracic endovascular aortic repair has rapidly supplanted conventional open repair because it is associated with lower morbidity and mortality rates. We report a case of SB-AAD and acute renal failure caused by renal malperfusion that was successfully treated with TEVAR and describe favourable aortic remodelling that was observed during 5-year follow-up.

## Summary figure

**Figure:**
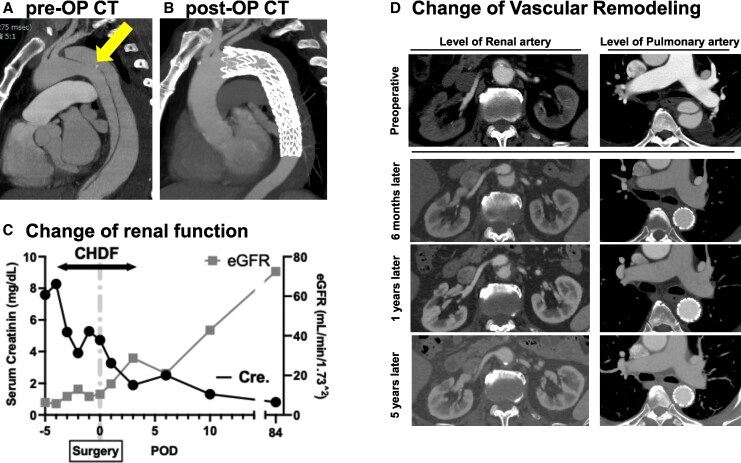


## Case presentation

**Figure 1:**
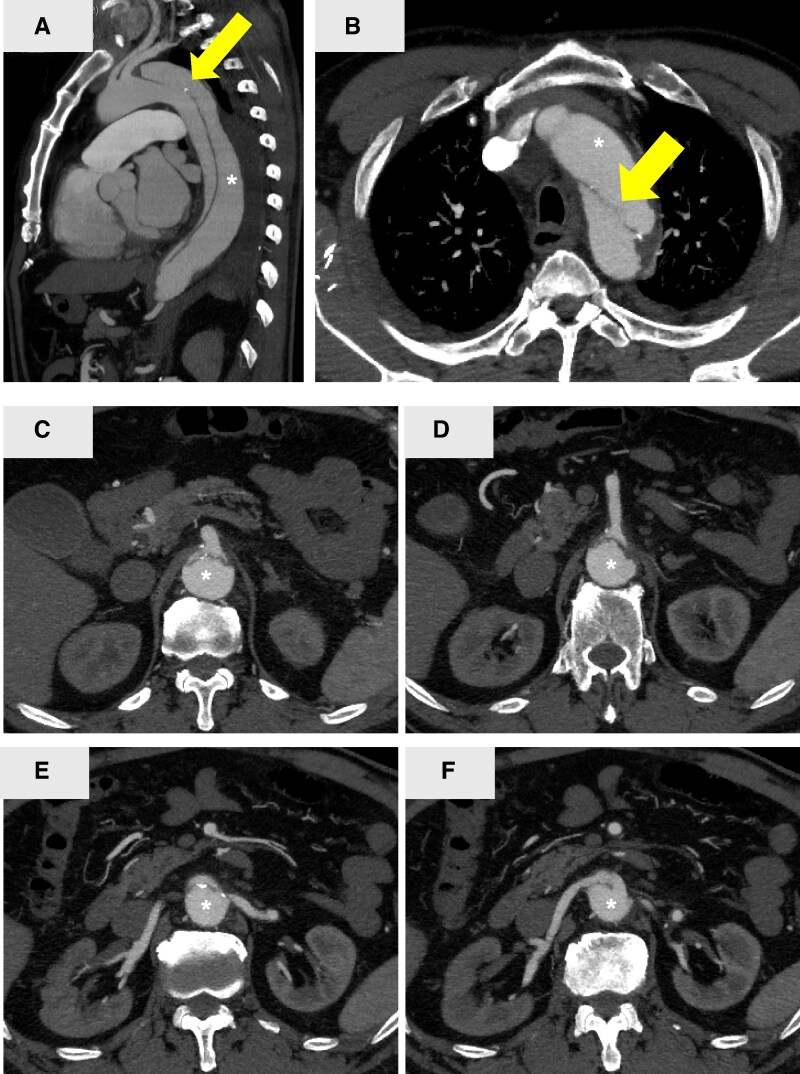
Preoperative enhanced computed tomography images of Stanford type B aortic dissection. An asterisk (*) indicates the false lumen. The primary entry tear is indicated by a yellow arrow in the sagittal (*A*) and axial (*B*) images of the distal aortic arch. The celiac artery (*C*), superior mesenteric artery (*D*), left renal artery (*E*), and right renal artery (*F*) branch from the true lumen. The density at the kidney in the early phase is lower than that at the aorta, suggesting renal ischaemia.

A 69-year-old man presented to a local hospital with chest pain. Enhanced computed tomographic angiography (CTA) revealed an entry tear (*Figure 1A, B*) at the distal aortic arch and dissection extending to the celiac artery. The patient was diagnosed with SB-AAD, and conservative management comprising pain relief and blood pressure control was initiated.

**Figure 2:**
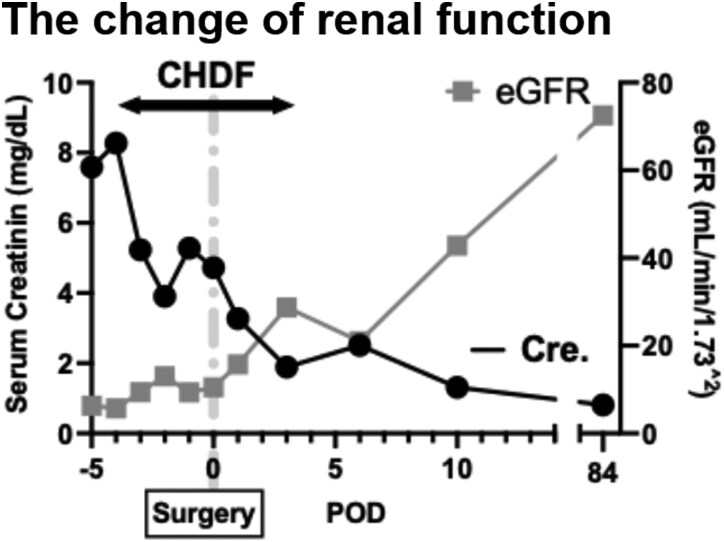
Change in the renal function. A closed circle indicates the serum creatinine level (mg/dL). A closed square indicates the change in the estimated glomerular filtration rate (mL/min/1.73 m^2^).

Eighteen days later, the patient’s renal function deteriorated and oliguria developed suddenly. Computed tomographic angiography revealed expansion of the dissection flap and false lumen distally and static compression of both renal arteries by the false lumen, resulting in renal ischaemia (*Figure 1E* and *F*). Abdominal visceral branches arose from the true lumen (*Figure 1C–F*); however, ischaemia was not observed in any other organs. Continuous haemodiafiltration was initiated for acute renal failure (*Figure 2*).

A detailed electrocardiogram-gated CTA analysis also identified the primary entry tear, which was less than 20 mm distal to the left subclavian artery on the greater curvature of the distal aortic arch (*Figure 1A* and *B*). Urgent TEVAR was planned to close the primary entry tear and ameliorate renal perfusion.

Under general anaesthesia, vascular access was achieved through the left common femoral artery. After systematic heparinization (activated clotting time > 250 s), a 0.035-in. guidewire and 4-Fr pigtail catheter were inserted through the true lumen to the ascending aorta via a 6-Fr introducer. Using transoesophageal echocardiography, particular attention was focused on the presence of the catheter in the true lumen. Thereafter, the guidewire was exchanged for a stiff guidewire, and a 24-Fr sheath was inserted. Additionally, a 4-Fr pigtail catheter was inserted via the right brachial artery for aortography.

**Figure 3:**
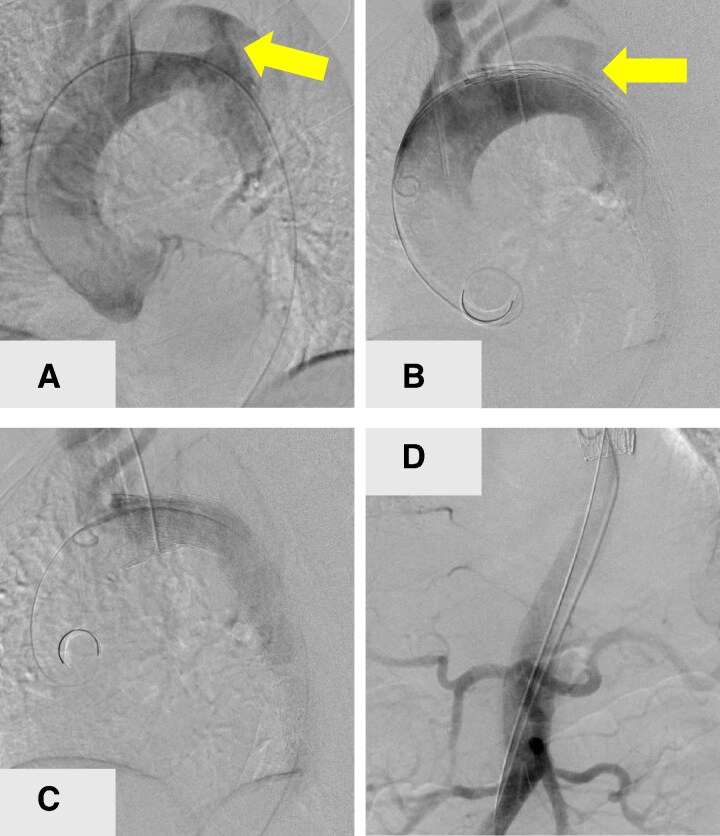
Perioperative aortography. The primary entry (yellow arrow) is recognized at the distal aortic arch (*A* and *B*). A proximal device is deployed from the orifice of the left subclavian artery (*B*). Leakage and related complications are not observed after deploying stent grafts (*C*). All abdominal vascular branches originate from the true lumen (*D*).

Two Gore-Tag stent grafts (W. L. Gore & Associates G.K., Flagstaff, AZ, USA) were deployed; a 28- × 100-mm stent graft was inserted in the true lumen of the descending aorta and a 37- × 150-mm stent graft was inserted proximally and extended from just below the left subclavian artery origin, overlapped by approximately 60 mm, and occluded the left subclavian artery after confirming retrograde blood flow from the right brachiocephalic artery to the left subclavian artery via the circle of Willis because the distance from the origin of the subclavian artery to the entry of aortic dissection was minimal (<2 cm) (*Figure 3A–C*). Intraoperative mean arterial pressure was maintained between 80 and 100 mmHg.

**Figure 4:**
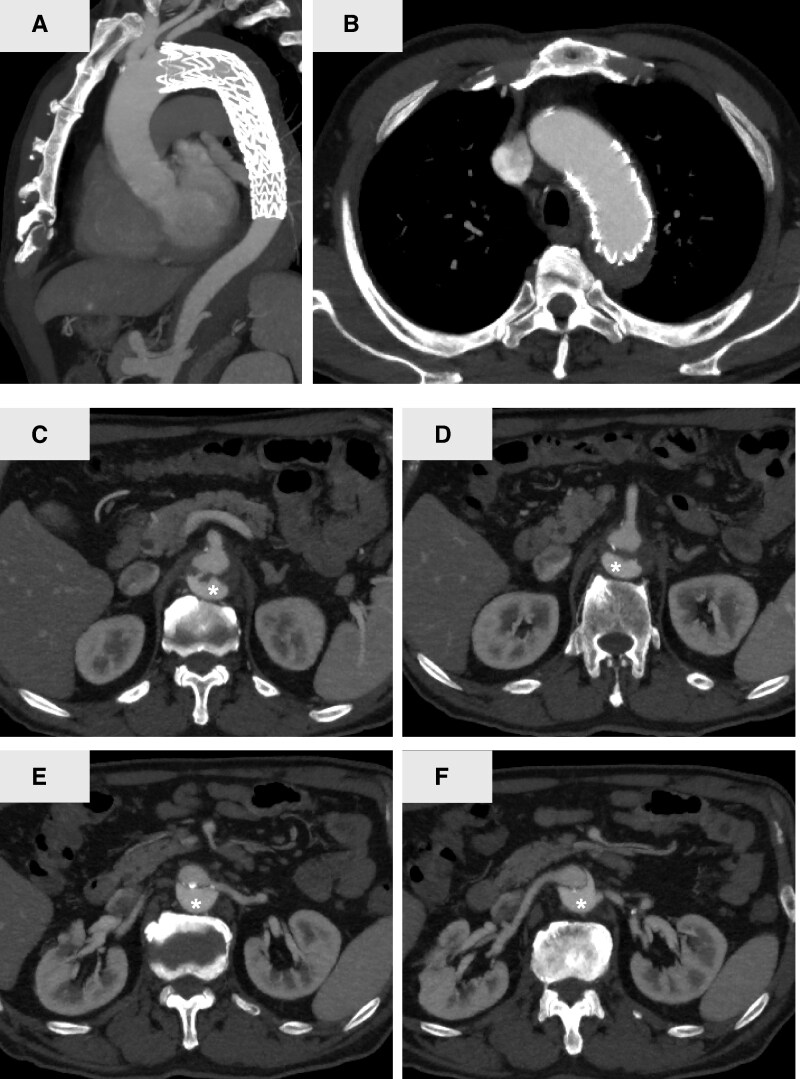
Enhanced computed tomography images at 14 days after thoracic endovascular aortic repair. Sagittal (*A*) and axial (*B*) images of the distal aortic arch. The celiac artery (*C*), superior mesenteric artery (*D*), left renal artery (*E*), and right renal artery (*F*) branch from the true lumen. The density at the kidney during the early phase is improved. An asterisk (*) indicates the false lumen.

Postoperative aortography confirmed the appropriate positioning of the stent graft (*Figure 3C*), re-expansion of the true lumen, and restoration of flow in both renal arteries (*Figure 3D*). Markedly increased urine output and renal function recovery were observed, and continuous haemodiafiltration was successfully weaned off (*Figure 2*). Follow-up CTA 2 weeks postoperatively revealed no enlargement of the dissection cavity and a well-expanded true lumen (*Figure 4A–F*). The patient was discharged on postoperative Day 14 without complications.

**Figure 5:**
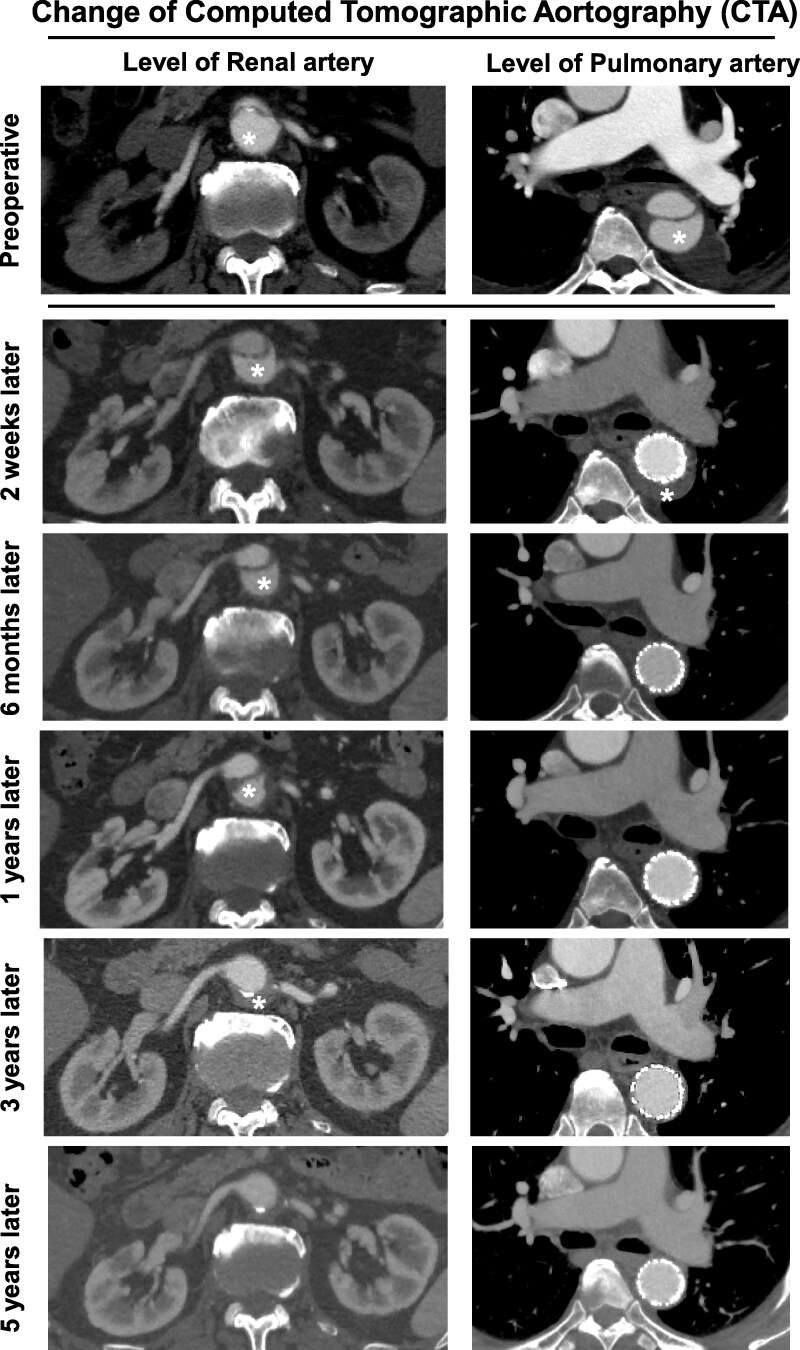
Preoperative and postoperative computed tomography findings. An asterisk (*) indicates the false lumen. (Left) Computed tomography images at the level of the renal artery. (Right) Computed tomography images at the level of the pulmonary artery.

Computed tomographic angiography was conducted during 5 years of follow-up and revealed optimal vascular remodelling of the aorta accompanied by complete disappearance of the false lumen (*Figure 5*). Aortic dissection recurrence and stent-related complications were not observed.

## Discussion

Patients with SB-AAD generally have better survival and a better prognosis than those of patients with Stanford type A aortic dissection. For SB-AAD without complications, conservative therapy comprising pain relief and blood pressure control is the initial treatment.^4,5^ However, SB-AAD with malperfusion is a risk factor for early death.^6,7^ Therefore, European Society of Cardiology (ESC) guidelines (class I, level B) indicate the need for surgical intervention at an early stage for SB-AAD with complications. Conventional open surgery and TEVAR are the surgical options for SB-AAD. Mortality and morbidity rates of patients who undergo TEVAR are better than those of patients who undergo conventional open surgery.^6,8^ European Society of Cardiology guidelines (class I, level B) also recommended TEVAR as a possible initial treatment.^5^

Our patient received conservative treatment during the acute phase of the first SB-AAD at a regional hub hospital staffed by cardiologists. Unfortunately, he experienced extension of the dissection flap and false lumen distally, which caused oliguria and acute renal failure that necessitated dialysis because of static compression of both renal arteries. Therefore, he was referred to our hospital for urgent surgical intervention. Because the preoperative CTA evaluation revealed that all abdominal vascular branches originated from the true lumen, closing the entry of the dissection using TEVAR decreased the pressure caused by the false lumen, expanded the true lumen, and improved renal artery blood flow without complications. Furthermore, during the 5-year follow-up period after TEVAR, the false lumen was absorbed and disappeared after thrombosis. Additionally, adequate vascular remodelling was observed.

Pre-emptive TEVAR may have effectively prevented malperfusion and renal failure if it had been performed earlier before re-dissection in this patient. Furthermore, pre-emptive TEVAR resulted in aortic remodelling and contributed to the prevention of aortic-related death caused by rupture.^9^ The optimal adaptation and timing of surgical intervention for SB-AAD without complications (specifically, whether it should be performed in the acute, subacute, or chronic phase after SB-AAD onset) are controversial.^10,11^ When performing TEVAR during the acute phase,^11^ when the dissected intima is fragile, the primary concern is the high risk of retrograde type A aortic dissection or stent graft-induced new entry tear. In contrast, when performing TEVAR during the chronic phase, a false lumen is more likely to persist postoperatively after closing the entry because the dissected intima can become thick and stable.^12^ Therefore, thorough discussions between cardiologist at a regional hub hospital and cardiovascular surgeons at a specialized centre regarding treatment planning are also important to enabling good outcomes for patients with SB-AAD without malperfusion.

Determining the optimal indication for and timing of TEVAR for SB-AAD during the acute or subacute phase is further complicated by the need to estimate the prognosis of the dissected cavity and possibilities of re-dissection and rupture. Consequently, the surgical timing and potential for complications attributable to surgical intervention must be considered when deciding how to proceed.

In summary, this case of complicated SB-AAD with renal malperfusion was successfully treated with TEVAR and resulted in favourable aortic remodelling during 5-year follow-up. Although surgical intervention must be carefully considered by surgeons and cardiologists, pre-emptive TEVAR during the early phase may be a safer and more effective treatment option for SB-AAD without malperfusion. Further case accumulation and investigation are required to determine the types of SB-AAD for which pre-emptive TEVAR is effective.

## Data Availability

The data that support the findings of this study are available from the corresponding author upon reasonable request. No restricted data were used in this study.

## Lead author biography

2000.06—Junior Resident, Tokushima University Hospital. 2002.6—Senior Resident, Division of Cardiovascular surgery, Tokushima University Hospital. 2006.04—Fellow, Division of cardiovascular surgery, EHIME Prefectural Central Hospital. 2008.04—Assistant Professor, Department of Cardiovascular Surgery, The University of TOKUSHIMA. 2011.07—Visiting Assistant Professor, Section of Cardiac Surgery, Yale University. 2012.09—Postdoctoral Researcher, Tissue Engineering Program and Surgical Research, Nationwide Children’s Hospital. 2008.04—Assistant Professor, Department of Cardiovascular Surgery, The University of Tokushima. 2019.04—Present Associate Professor, Department of Cardiovascular and Thoracic Surgery, Ehime University.

## Notes

**Consent:** Informed consent was obtained from the patient for the publication of this case report and accompanying images. No personal identifiers were included in this report to ensure the patient’s privacy and confidentiality. This study was conducted in accordance with institutional ethics guidelines and approved by the Institutional Review Board.
